# The effect of micropulse transcleral cyclophotocoagulation (MP-TSCPC) on anterior segment properties in glaucoma and ocular hypertension patients

**DOI:** 10.1007/s10792-025-03798-7

**Published:** 2025-10-28

**Authors:** Arij Daas, Ananth Ranjit, Thomas Sherman, Bhavin Patel, Elizabeth Galvis, Andrew Amon, Zoe Clarkson, Kin Sheng Lim

**Affiliations:** 1 Department of Ophthalmology, Guy’s and St Thomas’ NHS Foundation Trust, London, SE1 7EH UK; 2 Frost Eye Research Department, King’s College London, London, SE1 7EH UK; 3 KCL Frost Ophthalmology Research Department, St Thomas’ Hospital, Westminster Bridge Road, London, SE1 7EH UK

**Keywords:** Micropulse, Glaucoma, Ocular biomechanics

## Abstract

**Purpose:**

Micropulse Transcleral Cyclophotocoagulation (MP-TSCPC) lowers intraocular pressure by directing bursts of energy at the ciliary body which leads to a reduction in aqueous production. Although the diode laser radiation has a propensity for melanin in the ciliary epithelium, some energy dissipates into surrounding tissues. This study aims to investigate the ocular effects of MP-TSCPC on patients with glaucoma, focusing on corneal biomechanics, endothelial cell count, and refractive outcomes.

**Methods:**

Patients with open angle glaucoma and ocular hypertension were enrolled. Comprehensive ophthalmic investigations, including endothelial cell count, corneal topography, pupil diameter, and refraction, were conducted at baseline and six months post-MP-TSCPC.

**Results:**

Thirty-one eyes of 31 patients were included. At six months post-MP-TSCPC, a 2% decrease in corneal endothelial cell density was noted, though not statistically significant. A modest hyperopic shift in spherical equivalent was observed at 6 months and initially reached statistical significance. However, this did not remain significant following adjustment for multiple comparisons. Three patients had a significant hypermetropic shift. There was a statistically significant increase in pupil diameter.

**Conclusion:**

This is the first study to review various ocular parameters following micropulse transcleral cyclophotocoagulation. Overall, there was minimal clinically significant impact on ocular parameters.

## Introduction

The progressive and irreversible decline in vision observed in Open Angle Glaucoma (OAG) results from optic neuropathy, primarily mediated by elevated Intraocular Pressure (IOP) [1]. Treatment modalities for IOP pressure control include, reducing aqueous production, improving drainage through existing structures or providing an alternative drainage route. There are several cyclodestructive procedures, all of which employ a diode laser to target the ciliary process and associated vasculature to reduce aqueous production, thereby reducing IOP [2].

Since its inception, continuous wave transcleral diode laser (CW-TSCPC) has typically been reserved for refractory glaucoma and after other interventions have been exhausted [3]. This was due to unpredictable dose–effect relationship and reports of significant complications, which included hypotony, uveitis and reduced visual acuity [4–6]. Whilst retrospective studies suggest its use in non-refractory glaucoma, there is a lack of evidence in the form of randomised clinical trials to support its clinical utility in this patient group [7–9].

The predominant driver of the complications noted with (CW-TSCPC) is that the 810 nm infrared diode laser radiation, despite having a propensity for melanin pigment in the ciliary epithelium, is dissipated into surrounding tissue [10]. Micropulse Transcleral Cyclophotocoagulation (MP-TSCPC) theoretically circumvents this issue. In MP-TSCPC, the energy is delivered in short bursts to the targeted tissue (on-cycle), with the energy building up gradually to a photocoagulative state in the pigmented epithelium [11]. At the same time, the surrounding tissue can cool down during the off cycle, thereby theoretically minimising damage to surrounding tissue [11]. In a long term prospective clinical study, mean IOP showed a sustained reduction of 30–35% up to two years, with no serious complications noted in the post-operative period [12].

However, the scattering of the laser beam could potentially result in changes or damage to collateral tissue. Mastropasqua et al. noted that Ultrasonic Circular Cyclocoagulation (UCCC) induced anatomical changes within sclera and conjunctiva using Anterior Segment Optical Coherence Tomography (AS-OCT) [13]. Johnston et al. observed in an ex-vivo experiment that MP-TSCPC triggered ciliary muscle contraction, causing transient inward and posterior movement, as well as posterior displacement of the scleral spur and trabecular meshwork, altering Schlemm canal shape [14]. The recovery response of these structures to pre-laser treatment configuration was energy-dependent, with higher energy levels resulting in less recovery [14]. Scleral thinning and induced astigmatism have been reported in other cyclodestructive treatments [15, 16]. Mydriasis is a concern due to potential damage to the ciliary nerve following diode laser [17]. These collateral changes may have implications for the visual acuity and refractive outcomes post MP-TSCPC.

Another relevant tissue that may be implicated in collateral damage from MP-TSCPC is the corneal endothelium. The controversial withdrawal of CyPass Micro-Stent (Alcon, Geneva, Switzerland) from the global market based on the results of the COMPASS-XT study which demonstrated significant endothelial cell loss has highlighted this safety concern in all new forms of glaucoma treatment [18]. To date, none of these potential changes to the biomechanics and other tissue effects following MP-TSCPC have been comprehensively studied. We aim to investigate these through a case–control observational study involving individuals diagnosed with open-angle glaucoma (OAG) and ocular hypertension (OHT).

## Methods

Patients with open angle glaucoma (OAG) or ocular hypertension (OHT) were invited to participate in the study. Open angle glaucoma cases included any cases of POAG or pigmentary glaucoma, diagnosed by a glaucoma subspecialist in their standard care glaucoma clinic at St Thomas’ Hospital, London, United Kingdom.

A patient information leaflet was provided at initial contact, and a signed consent form was obtained before the measurements and treatment were carried out.

Ethics approval was obtained from the National Health Service (NHS) research ethics committee. The research conformed to the Declaration of Helsinki.

### Eligibility criteria

The following inclusion criteria had to be met to be included in the study:

- Patients between 18 and 90 years old.
- Willing, able and available to participate in all aspects of the study, including understanding the study and giving informed consent.
- Diagnosis of OAG or OHT with suboptimal IOP despite maximal tolerated medical treatment (including selective laser trabeculoplasty).

Patients were excluded if they had any of the following:

- Cognitive impairment conflicting with informed consent or follow up
- Current participation in any clinical trial
- Allergy to fluorescein
- Previous intraocular incisional operations

All patients underwent a comprehensive ophthalmic examination which included Visual Acuity (VA) in logMAR, slit lamp examination, gonioscopy and dilated fundal examination.

Anterior chamber depth (ACD) and axial length (AXL) measured with the IOL Master (Carl Zeiss Meditec, Dublin, CA) in automatic mode under standard clinical photopic conditions, with patient fixating on internal target. Measurements were accepted if the device indicated adequate signal quality and proper alignment. The automatically averaged value from the IOL Master was used for analysis.

Corneal hysteresis (CH) and corneal resistance factor (CRF) assessed using the Ocular Response Analyzer (ORA, Reichert, New York). Four readings were obtained per eye and measurements with poor waveform scores were excluded. The mean of valid readings was used for analysis.

Endothelial cell count (ECC) and coefficient of variation (CV) determined with the Tomey EM-3000 specular microscope (Tomey, Nagoya, Japan). At least 100 cells were analysed per image in automatic mode and the best quality scan was used.

Corneal topography (K1, K2, Kmax, thinnest corneal thickness) obtained using the Oculus Pentacam (Pentacam 70700, Oculus, Wetzlar, Germany). Measurements were performed under standard clinical photopic conditions with patients fixating on the internal target. A quality rating score is provided, and the best quality scan was used.

Central corneal thickness (CCT) measured with the Pachmate DGH 55 (DGH Technology Inc., Exton, PA). An automated system provides an average and standard deviation with appropriate applanation.

Pupil diameter was measured under low light conditions, with the patient focusing on a distant object. A manual approach was utilised to control for accommodation. Measurements were taken separately for the right and left eyes using pupil cards and a pen torch, which was directed obliquely into the anterior chamber.

Manifest refraction was conducted with trial frames and lenses in photopic conditions. The patient was positioned 3 m from the test chart and all refractions were performed by an optometrist. The refraction process consisted of an adjustment of the spherical power, followed by cylinder axis and power, with a final refinement of spherical power. This was followed by the addition of plus power lenses to establish the near add (ADD). Each eye was measured separately with the fellow eye being occluded. Visual acuity (VA) was recorded following refraction.

Amplitude of accommodation (AMP-ACC) was measured monocularly using push-up and push-down methods. Patients wore their full distance correction determined during manifest refraction. The patient was asked to look at the N5 line on Royal Air Force (RAF) rule at 40 cm and the target was slowly moved towards the patient’s fixating eye until the patient noticed the first signs of blurring. While the target was brought closer to the patient, they were continuously asked if the target remained clear and were reminded to report when the target started to become blurry. A reading was taken when the patient first noticed the target become blurry. The push up method was performed by pushing the target towards the patient until it was blurry, then pulled away until the patient first noticed the target was clear. The average of the two was taken as the amplitude of accommodation. A reading in dioptres was taken from the RAF rule.

These measurements were taken at baseline and six months post-laser. Both the operated eye and the fellow untreated eye were assessed, with the latter serving as a control.

### MP-TSCPC treatment procedure

MP-TSCPC laser treatment was performed at an ambulatory surgical centre by one surgeon. The original MicroPulse P3 Probe was used, which has now been discontinued. Peribulbar anaesthesia (a mixture of 3 ml of Lidocaine 2% + 2 ml of Levobupivacaine) was performed by the surgeon. A speculum was used pre-procedure. Lubricating gel was used to maintain a wet field during the laser procedure. The probe was placed firmly against the conjunctiva avoiding the sclera and sparing 3 and 9 o’clock position. The probe tip was positioned parallel to the surface, 3 mm from the limbus.

The procedure adhered to the following parameters: power settings were set to 2000 mW, with a duty cycle of 31.3% and 4–5 sweeps. Pulses were administered over 80 s in both the superior and inferior quadrants. All patients received a subconjunctival steroid injection.

### Post MP-TSCPC treatment

The eye treated with MP-TSCPC was treated topically with Dexamethasone 0.1% preservative free 4 times a day for 1 week then twice a day for 1 week.

Patients were reviewed at 1 day, 1 week, 1 month, 3 months, 5 months, and 6 months postoperatively.

### Statistical analysis

This study was conducted alongside the primary research that investigated the effects of MP-TSCPC on aqueous dynamics. Consequently, the sample size for this study was determined based on the need to detect differences in two key parameters: aqueous flow and outflow facility. This was informed by an earlier study conducted at the Mayo Clinic in Rochester, MN, USA, led by the chief investigator (KSL) [19]. The earlier study demonstrated that 30 subjects would be required to achieve a 90% probability of detecting a 5% change in IOP, a 5.4% change in aqueous flow, and a 7.5% change in outflow facility between medication groups, if such differences existed, with an alpha of 0.05 and a beta of 0.10.As such the findings should be interpreted as exploratory and underpowered for small structural/biometric effects.

Histograms and Shapiro–Wilk tests were utilized to assess the normality of data distribution. A Shapiro–Wilk W value greater than 0.05 indicated a normal distribution. Continuous variables were compared using paired t-tests, including baseline comparisons between the treated and fellow untreated eyes. When normality assumptions were not met, the Wilcoxon signed-rank test was applied. A p-value less than 0.05 was considered statistically significant. To control for false discovery rate, an adjusted p value was calculated utilising Benjamini–Hochberg procedure. All analyses were performed using SPSS version 25.0 (SPSS, Chicago, IL).

## Results

Forty eligible patients were invited to enrol in the study and 9 patients declined to participate. Thirty-one eyes of 31 patients were included at baseline, with the fellow eyes of recruited patients, which had not undergone any surgical intervention prior to or during the study, forming the control group. Baseline characteristics are described in Table 1.

**Table 1:** Baseline characteristics of treated and control eyes

	Treated eyes (n = 31) Mean ± SD	Fellow untreated eyes (n = 31) Mean ± SD
Age (years)	63 ± 8.2	63 ± 8.2
Race (Black:White)	22:9	22:9
Diagnosis (OHT:OAG:PDG)	5:25:1	11:19:1
Sex (Female:Male)	12:19	12:19
VA (Logmar)	0.002 ± 0.08	0.03 ± 0.18
AXL (mm)	24.19 ± 1.62	24.31 ± 1.26

OHT: ocular hypertension, OAG: open angle glaucoma, PDG: pigment dispersion glaucoma, VA: best corrected visual acuity, AXL: Axial Length

### Baseline parameters

The study population comprised of predominantly black (n = 22, 71%) and male (n = 19, 61.2%) patients. Average age of participants was 63 (± 8.2) and primary open-angle glaucoma was the main diagnosis. No significant differences existed between the control eyes and treated eyes with respect to baseline characteristics.

Corneal endothelial cell density, central corneal thickness, and coefficient of variation of cell size are shown in Table 2. There was no statistically significant difference in all the parameters between the treated and control group.

**Table 2:** Baseline of the corneal topography, corneal properties and corneal endothelial cell characteristics in the treated and fellow untreated eyes

	Treated eyes (n = 31) Mean ± SD	Fellow Untreated Eyes (n = 31) Mean ± SD	*P* value	Adjusted *P* values	Effect size (95% Confidence Interval)
ACD (mm)	3.28 ± 0.33	3.29 ± 0.35	0.4	0.9	0.01 (− 0.03 to 0.07)
PD (mm)	3.13 ± 0.6	3.13 ± 0.6	1.0	1.00	0.00 (0.00 to 0.00)
ECC (cells/mm^2^)	2457 ± 275.67	2452 ± 207.23	0.9	0.99	− 5 (− 67.52 to 73.68)
CV (%)	41.04 ± 8.0	41.20 ± 12.41	0.9	0.99	0.16 (− 5.83 to 6.15)
CCT (µm)	529.2 ± 30.17	529.0 ± 28.52	0.5	0.9	− 0.2 (− 2.72 to 5.57)
Kmax (D)	44.65 ± 1.46	44.70 ± 1.82	0.7	0.9	0.05 (− 0.53 to 0.38)
TL (µm)	522.90 ± 25.25	519.17 ± 28.14	0.3	0.9	− 3.73 (− 14.22 to 4.22)
CH (mmHg)	7.46 ± 3.32	7.36 ± 2.12	0.9	0.99	− 0.1 (− 1.46 to 1.29)
CRF (mmHg)	11.28 ± 2.97	10.41 ± 1.43	0.2	0.9	− 0.87 (− 2.18 to 0.46)

ACD: anterior chamber depth, PD: pupil diameter, ECC: endothelial cell count, CV: coefficient of variation of endothelial cell size, CCT: central corneal thickness, Kmax: maximum curvature power of the front surface of the cornea, TL: thinnest corneal location, CH: corneal hysteresis, CRF: corneal resistance factor

ACD measured with IOL Master (Carl Zeiss Meditec, Dublin, CA); PD measured under low-light conditions using pupil cards and pen torch; ECC and CV measured with Tomey EM-3000 specular microscope (Tomey, Nagoya, Japan); corneal topography (Kmax and thinnest location) measured with Pentacam (Oculus, Wetzlar, Germany); CCT measured with Pachmate DGH 55 (DGH Technology Inc., Exton, PA); CH and CRF measured with Ocular Response Analyzer (ORA, Reichert, New York)

The baseline characteristics of the manifest refraction, addition of plus power (ADD) for near vision and the amplitude of accommodation (AMP-ACC) are shown in Table 3. Ocular Response Analyser parameters including CH and CRF are shown in Table 2. There was no statistically significant difference in all the parameters between the treated and controlled group.

**Table 3:** Refractive error baseline characteristics

	Treated eyes (n = 22) Mean ± SD	Fellow untreated eyes (n = 22) Mean ± SD	*P* value	Adjusted *P* value	Effect size (95% Confidence Interval)
SE (D)	− 0.91 ± 3.40	− 0.59 ± 3.35	0.2	0.4	0.4 (− 0.24 to 0.89)
Sphere (D)	− 0.60 ± 3.36	− 0.24 ± 3.21	0.1	0.4	0.36 (− 0.11 to 0.84)
Cylinder (D)	− 0.63 ± 0.56	− 0.69 ± 0.58	0.6	0.6	− 0.06 (− 0.32 to 0.19)
AMP-ACC (D)	1.57 ± 0.73	1.65 ± 0.71	0.3	0.4	0.08 (− 0.08 to 0.2)
ADD (D)	1.72 ± 0.56	1.72 ± 0.56	–		–

Manifest refraction was performed with trial frames and lenses in photopic conditions at a 3 m fixation distance, without cycloplegia. Amplitude of accommodation was measured monocularly using the Royal Air Force (RAF) rule with push-up and push-down methods, with patients wearing full distance correction

SE = spherical equivalent, AMP-ACC = amplitude of accommodation, ADD = near add

### Baseline—6 month parameters

Of the 31 patients enrolled in the study, at six months post- MP-TSCPC, only 28 patients were invited to attend the 6 months follow up visit, as 3 cases had further intraocular surgeries during the first 6 months.

There was a statistically significant change in ACD from baseline to 6 months in the controlled eyes, though this was not clinically significant and significance did not remain after Benjamini-Hochberg (BH) procedure. There was no significant change in any of the other parameters in the controlled eyes, between baseline and 6 months (Tables 4 and 5).

**Table 4:** Fellow Untreated Eye:ECC, central corneal thickness, cell size coefficient of variation, and corneal topography at 6 months

	Baseline (n = 31) Mean ± SD	6 Months (n = 28) Mean ± SD	*P* value	Adjusted *P* values	Effect size (95% Confidence Interval)
ACD (mm)	3.29 ± 0.35	3.28 ± 0.33	0.02	0.2	− 0.01 (− 0.011 to − 0.01)
PD (mm)	3.13 ± 0.61	3.28 ± 0.56	0.06	0.3	0.15 (− 0.004 to 0.30)
ECC (cells/mm^2^)	2452 ± 207.23	2440 ± 258.80	0.9	0.9	− 12 (− 65.21 to 65.93)
CV (%)	41.20 ± 12.41	39.30 ± 6.12	0.3	0.5	− 1.9 (− 7.51 to 2.33)
CCT (µm)	529.0 ± 28.52	529.81 ± 31.06	0.4	0.5	0.81 (− 8.12 to 3.62)
Kmax (D)	44.70 ± 1.82	44.61 ± 1.68	0.5	0.6	− 0.09 (− 0.32 to 0.15)
K1(D)	43.09 ± 1.65	42.94 ± 1.48	0.2	0.5	− 0.15 (− 0.80 – 0.20)
K2 (D)	43.69 ± 1.59	43.76 ± 1.55	0.3	0.5	0.07 (− 0.06 to 0.17)
Thinnest local (µm)	519.17 ± 28.14	512.36 ± 27.51	0.2	0.5	− 6.81 (− 14.05 to 2.36)
CH (mmHg)	7.36 ± 2.12	7.54 ± 2.06	0.4	0.5	0.18 (− 1.31 to 0.60)
CRF (mmHg)	10.41 ± 1.43	11.23 ± 5.48	0.3	0.5	0.82 (− 1.36 to 4.16)

ACD measured with IOL Master (Carl Zeiss Meditec, Dublin, CA); PD measured under low-light conditions using pupil cards and pen torch; ECC and CV measured with Tomey EM-3000 specular microscope (Tomey, Nagoya, Japan); corneal topography (K1, K2, Kmax, thinnest location) measured with Pentacam (Oculus, Wetzlar, Germany); CCT measured with Pachmate DGH 55 (DGH Technology Inc., Exton, PA); CH and CRF measured with Ocular Response Analyzer (ORA, Reichert, New York)

ACD: anterior chamber depth, PD: pupil diameter, ECC: endothelial cell count, CV: coefficient of variation of endothelial cell size, CCT: central corneal thickness, Kmax: maximum curvature power of the front surface of the cornea, K1: flattest meridian of the cornea, K2: steepest meridian of the cornea, TL: thinnest corneal location, CH: corneal hysteresis, CRF: corneal resistance factor

**Table 5:** Refractive error at 6 months in fellow untreated eyes

	Baseline (n = 22)* Mean* ± *SD*	6 months (n = 22)* Mean* ± *SD*	*P* value	Adjusted *P* value	Effect Size 95% Confidence Interval
SE (D)	− 0.59 ± 3.35	− 0.65 ± 3.36	0.2	1.0	− 0.06 (− 0.16 to 0.13)
Sphere (D)	− 0.24 ± 3.21	− 0.26 ± 3.22	0.7	1.0	− 0.02 (− 0.13 to 0.17)
Cylinder (D)	− 0.69 ± 0.58	− 0.77 ± 0.59	0.1	0.3	− 0.08 (− 0.18to 0.02)
AMP-ACC (D)	1.65 ± 0.71	1.75 ± 0.82	0.99	0.99	0.1 (− 0.42 to 0.42)
ADD (D)	1.72 ± 0.56	2.04 ± 0.46	0.07	0.4	0.32 (− 0.03 to 0.64)

Manifest refraction was performed with trial frames and lenses in photopic conditions at a 3 m fixation distance, without cycloplegia. Amplitude of accommodation was measured monocularly using the Royal Air Force (RAF) rule with push-up and push-down methods, with patients wearing full distance correction

SE: Spherical Equivalent, AMP-ACC: amplitude of accommodation, ADD: near add

At 6 months CH, CRF, AMP-ACC and ACD were not significantly changed after MP-TSCPC. There was a 2% decrease in ECC in the treated eye as shown in Table 5 compared with 0.5% in the untreated eye. However, this was not statistically significant (Table 6).

**Table 6:** Study Eye ECC, anterior chamber depth, central corneal thickness and cell size coefficient of variation, corneal topography before and after MP-TSCPC

	Baseline (n = 31) Mean ± SD	6 months (n = 28) Mean ± SD	*P* value	Adjusted *P* value	Effect Size 95% Confidence Interval
ACD (mm)	3.28 ± 0.33	3.29 ± 0.31	0.3	0.4	0.01 (− 0.07 to 0.02)
PD (mm)	3.13 ± 0.60	3.70 ± 0.66	< 0.00001*	0.0001	0.57 (0.32 to 0.82)
ECC (cells/mm2)	2457 ± 275.7	2415 ± 220.0	0.3	0.4	− 42 (− 121.9 to 42.55)
CV (%)	41.04 ± 8.0	40.43 ± 8.04	0.9999	0.99	− 0.61 (− 3.83 to 3.83)
CCT (µm)	529.2 ± 30.17	526.9 ± 32.28	0.3	0.4	− 2.3 (− 8.76 to 3.14)
Kmax (D)	44.65 ± 1.46	44.48 ± 1.66	0.1	0.2	− 0.17 (− 0.54 to 0.09)
K1 (D)	43.23 ± 1.53	43.01 ± 1.60	0.1	0.2	− 0.22 (− 0.67 to 0.08)
K2 (D)	43.9 ± 1.43	43.74 ± 1.63	0.2	0.4	− 0.16 (− 0.56 to 0.14)
Thinnest local (µm)	522.9 ± 25.25	511.9 ± 34.30	0.03*	0.2	− 11 (− 22.64 to − 1.43)
CH (mmHg)	7.46 ± 3.32	7.47 ± 2.20	0.6	0.7	0.01 (− 2.74 to 1.54)
CRF (mmHg)	11.28 ± 2.97	10.17 ± 2.32	0.1	0.2	− 1.11 (− 3.02 to 0.46)

ACD measured with IOL Master (Carl Zeiss Meditec, Dublin, CA); PD measured under low-light conditions using pupil cards and pen torch; ECC and CV measured with Tomey EM-3000 specular microscope (Tomey, Nagoya, Japan); corneal topography (K1, K2, Kmax, thinnest location) measured with Pentacam (Oculus, Wetzlar, Germany); CCT measured with Pachmate DGH 55 (DGH Technology Inc., Exton, PA); CH and CRF measured with Ocular Response Analyzer (ORA, Reichert, New York)

ACD: anterior chamber depth, PD: pupil diameter, ECC: endothelial cell count, CV: coefficient of variation of endothelial cell size, CCT: central corneal thickness, Kmax: maximum curvature power of the front surface of the *cornea, K1:* flattest meridian of the cornea, K2: steepest meridian of the cornea, TL: thinnest corneal location, CH: corneal hysteresis, CRF: corneal resistance factor

**Fig. 1:**
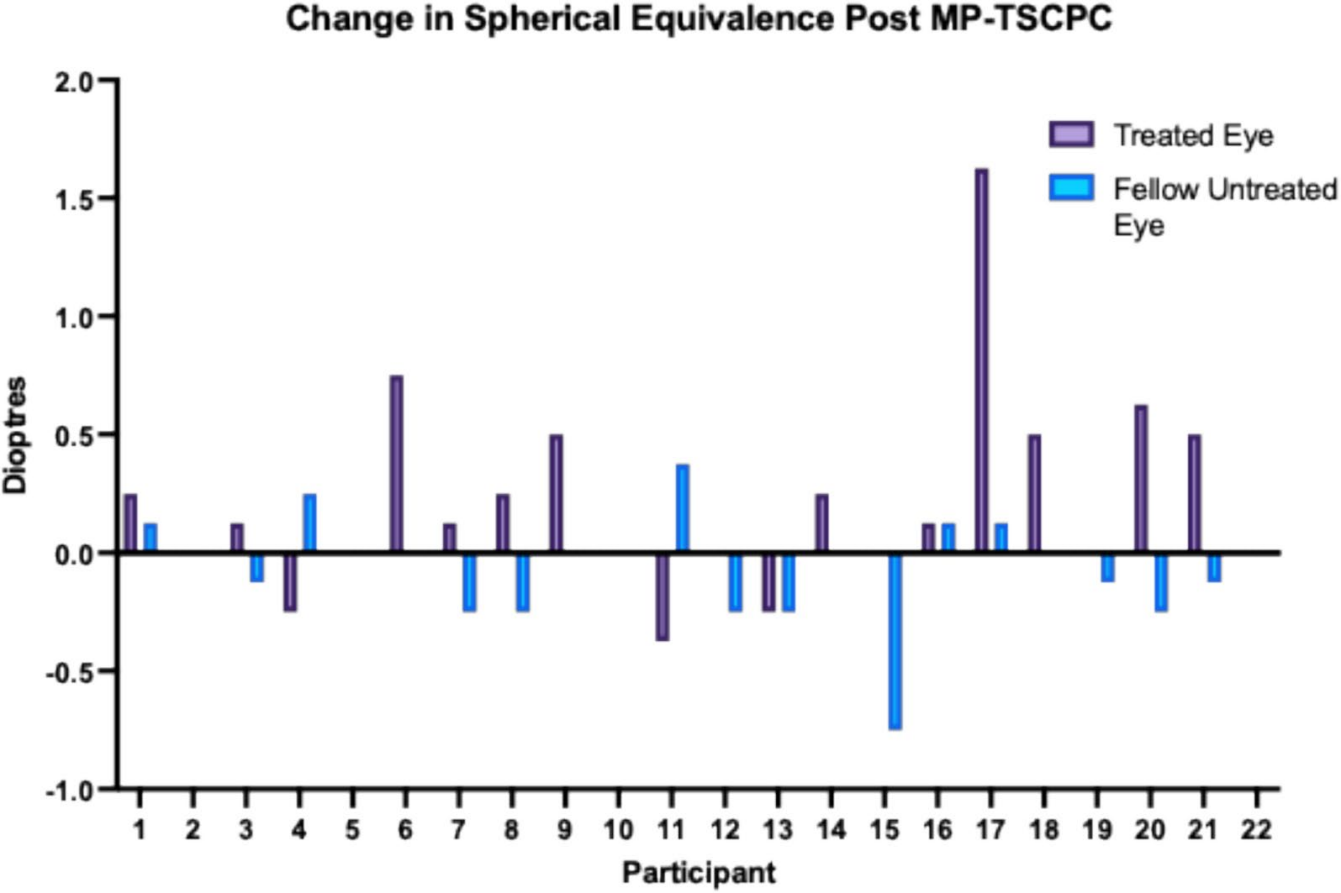
Change in Spherical Equivalence Post MP-TSCPC

In the treated eyes, there was a small but statistically significant hyperopic shift in spherical equivalent from baseline to 6 months (mean change + 0.21 D, 95% CI 0.03–0.41, *p* = 0.03). A similar effect was observed in spherical power (+ 0.25 D, 95% CI 0.04–0.46, *p* = 0.02). However, neither association remained statistically significant after controlling for the False Discovery Rate (FDR) using the Benjamini–Hochberg (BH) procedure. A sensitivity analysis of spherical equivalent data was performed excluding four identified outliers. The mean hyperopic shift in spherical equivalent was + 0.24 D, 95% CI 0.03–0.46, *p* = 0.03. An individual-level waterfall plot of change in spherical equivalent for treated vs control eyes is shown in Fig. 1. Three patients demonstrated hypermetropic shifts in spherical equivalent of + 0.63 D, + 0.75 D, and + 1.63 D, with corresponding ACD changes of + 0.09 mm,  − 0.04 mm, and + 0.13 mm, respectively.

A statistically significant difference was observed in Thinnest corneal Location (TL) at 6 months, with a decrease of 2.1% (522.9 ± 25.25 vs 511.9 ± 34.30, *p* = 0.03). Though, this did not persist after adjustment with BH procedure. As shown in Table 7, there was a statistically significant change in pupil diameter following the laser treatment, which remained after adjustment with BH procedure.

**Table 7:** Refractive error before and after MP-TSCPC

	Baseline (N = 22) Mean ± SD	6 months (N = 22) Mean ± SD	*P* value	Adjusted *P* value	Effect size, 95% Confidence Interval
SE (D)	− 0.91 ± 3.40	− 0.70 ± 3.45	0.03*	0.08	0.21 (0.03 to 0.41)
Sphere (D)	− 0.60 ± 3.36	− 0.35 ± 3.37	0.02*	0.08	0.25 (0.04 to 0.46)
Cylinder (D)	− 0.63 ± 0.56	− 0.69 ± 0.63	0.3	0.4	− 0.06 (− 0.07 to 0.21)
AMP-ACC (D)	1.57 ± 0.73	1.64 ± 0.84	0.8	0.8	0.07 (− 0.48 to 0.39)
ADD (D)	1.72 ± 0.56	2.03 ± 0.44	0.08	0.1	0.31 (− 0.03 to 0.62)

Manifest refraction was performed with trial frames and lenses in photopic conditions at a 3 m fixation distance, without cycloplegia. Amplitude of accommodation was measured monocularly using the Royal Air Force (RAF) rule with push-up and push-down methods, with patients wearing full distance correction

SE: Spherical Equivalent, AMP-ACC: amplitude of accommodation, ADD: near add

## Discussion

The study involved 31 patients with primary open-angle glaucoma, predominantly black and male, with their fellow eyes forming the control group. Baseline characteristics showed no significant differences between treated and fellow untreated eyes. Parameters such as corneal endothelial cell density, central corneal thickness, and manifest refraction at baseline did not differ significantly between the groups. At 6 months, there were no clinically significant changes in control eyes. In treated eyes, there was a 2% decrease in corneal endothelial cell density, though not statistically significant. Though a statistically significant change in spherical equivalent and sphere was identified in treated patients, this did not remain after controlling for the false discovery rate using the Benjamini–Hochberg procedure. There was a statistically significant decrease in mean local thinnest corneal thickness, though again was insignificant after adjustment with BH. Additionally, there was a statistically significant change in pupil diameter following laser treatment.

To our knowledge, our study is the first ever to assess the changes in ocular properties such as corneal biomechanics and refraction following MP-TSCPC in patients with OHT or mild to moderate OAG. The study mitigated the impact of glaucoma drops on ocular properties as all patients underwent one month of washout at baseline and six months after having MP-TSCPC.

In the treated eyes, we observed a statistically significant reduction in thinnest corneal location (TL) at 6 months (522.9 ± 25.25 vs. 511.9 ± 34.30 µm, *p* = 0.03), though this finding did not persist after correction for multiple comparisons using the Benjamini–Hochberg procedure. Though speculative, thinning of the local corneal area may be attributed to inadvertent damage to the long ciliary nerves, which contribute to innervation of the anterior segment, even when the 3 and 9 o’clock meridians are carefully avoided. Perez et al. suggest that the MP3 probe’s size and curved contact point could affect the perilimbal nerve plexus, a structure comprising branches of the long ciliary nerve [20]. Additionally, the continuous ‘painting motion’ employed could induce mechanical injury to the perilimbal corneal plexus [6]. This risk is heightened when the probe is positioned too anteriorly to the cornea. Despite taking care to avoid the 3 and 9 o'clock positions and over-treatment, MP-TSCPC led to two cases of relapsing neurotrophic keratitis one month after laser treatment and a similar case has also been reported by Kim et al. [20, 21]. Within our cohort of patients there were no reports of dry eye symptoms or neurotrophic keratitis. Though, it is important in the context of this exploratory study to consider other factors which may impact TL, such as diurnal effects and steroid exposure post MP-TSCPC in the treated eye.

A statistically significant increase in pupil diameter was observed in our cohort of patient receiving MP-TSCPC, when measured under low light conditions. Although no cases of fixed dilated pupils were recorded, other studies on Micropulse Transscleral Cyclophotocoagulation have reported this phenomenon, which resolved over time [22, 23]. Though the impact on pupil diameter may be a consequence of various factors, those suggested include a transient rise in IOP causing iris vasculature occlusions with subsequent ischaemia [22].

It is appreciated within the literature that the yearly reduction in endothelial cell count is by 0.3–0.5% [11, 12]. The study demonstrated a 2% decrease in ECC in the treated eyes compared to 0.05% in the untreated eye. However, it was not statistically significant and Kuebler et al. have similarly reported no significant difference from baseline to 6 months [24]. Our study did not observe any statistically significant changes in central corneal thickness post MP-TSCPC. This contrasts with transcleral contact diode laser cyclophotocoagulation where a significant increase has been noted in the 10-day post-procedural period and suggested to be due to inflammation [25]. The pulsatile nature of micro-pulse, whereby energy builds up to a photocoagulative state in pigmented epithelium with associated off period to allow surrounding tissue to cool down may minimise the level of inflammation that occurs.

Manifest refraction was performed on 22 patients during the study period. Although an apparent hyperopic shift in mean spherical power and spherical equivalent was observed at 6 months, statistical significance was not sustained after adjustment for multiple comparisons using the Benjamini–Hochberg procedure. Three patients demonstrated hypermetropic shift in spherical equivalent of + 0.63 D, + 0.75 D, and + 1.63 D, with corresponding ACD changes of + 0.09 mm,  − 0.04 mm, and + 0.13 mm, respectively. Although posterior displacement of the irido-lenticular diaphragm could theoretically induce a hyperopic shift, the small magnitude and inconsistent direction of ACD changes observed do not correlate with the refractive changes. Scleral thinning has been documented following MP-TSCPC and the potential corneal flattening stemming from these alterations might play a role in the observed hyperopic shift [26, 27]. However, given the exploratory nature of this analysis, and the fact that refraction was not a predefined study endpoint, these findings should be interpreted with caution. Moreover, because manifest refraction was used, small accommodation effects in presbyopic patients may have impacted results. Further studies, which include imaging such as Anterior Segment Optical Coherence Tomography (AS-OCT) are required to investigate corneal changes and confirm refractive changes. Of note there are no other studies which have investigated refractive shift with respect to MP-TSCPC.

The 6-month duration of the study limits the ability to appreciate whether there will be any long-term impacts on the cornea or whether any of the changes such as the mean thinnest local cornea thickness persist. Despite no patients reporting any dry eye symptoms, it would be important to assess if there were any changes to the tear film due to MP-TSCPC. The treated eye received intra-operative steroid injection alongside a 2-week course of topical steroids which may influence long-term ocular parameters. Pupil diameter was measured manually rather than with an automated device such as the Pentacam. While this allowed control for accommodation during measurement, it may have introduced measurement variability. Additionally, we acknowledge that ocular parameters were not standardised to a particular time of day and diurnal variation may have impacted the values obtained. Given the limited number of studies evaluating MP-TSCPC and its impact on ocular parameters, these results provide valuable early insights. However, it is acknowledged that future studies are required to appreciate their wide implications.

It should be noted that our study utilised the original MicroPulse P3 probe, which has since been discontinued. In 2020, Iridex introduced a revised MicroPulseP3 with several features to improve delivery and safety [28]. Key changes include delivery of laser energy more posterior to the limbus, a recessed tip, and a concave baseplate that enhances scleral stability and minimises the anterior displacement seen with the original MicroPulse [29]. Thereby, theoretically reducing direct exposure of the perilimbal nerve plexus, limbal vasculature and goblet cells, thereby reducing risk of ocular surface disease and anterior segment changes [30]. Regardless of probe design, treatment duration of MP-TSCPC can vary significantly amongst clinicians from 40 to 100 s and whilst 2000mW are typically used, it is up to the discretion of the surgeon whether they use higher energy settings [6].

Though MP-TSCPC has seen utility in clinical settings outside the UK, the National Institute for Health and Care Excellence (NICE) has suspended its use within clinical practice, and it is reserved primarily for research [31]. This outcome was a result of the quality of evidence on efficacy being deemed inadequate, citing lack of randomised controlled studies and lack of clarity regarding patient selection. Whilst the efficacy of this procedure is contested, further research should include an assessment of key ocular parameters such as manifest refraction and ECC. Standardisation of practice continues to be an issue for the procedure itself but also its place in the glaucoma treatment pathway.

## Conclusion

This is the first study to comprehensively review a variety of ocular parameters in the context of micropulse transcleral cyclophotocoagulation. As highlighted elsewhere in the literature this procedure has a significantly better safety profile compared with continuous wave transcleral diode laser, which is more commonly used within clinical practice. As the utility of micropulse transcleral cyclophotocoagulation remains in question, it is important to consider the impact of this procedure on ocular parameters in subsequent research.

### Clinical significance

Some patients experienced significant hypermetropic shift and this risk should be conveyed when counselling patients. Overall, Micropulse Transcleral Cyclophotocoagulation had minimal impact on ocular parameters.

## Data Availability

No datasets were generated or analysed during the current study.
